# Dose Reduction of Dynamic Computed Tomography Myocardial Perfusion Imaging by Tube Voltage Change: Investigation in a Swine Model

**DOI:** 10.3389/fcvm.2022.823974

**Published:** 2022-03-03

**Authors:** Wenlei Geng, Yang Gao, Na Zhao, Hankun Yan, Wei Ma, Yunqiang An, Liujun Jia, Bin Lu

**Affiliations:** ^1^Department of Radiology, Fuwai Hospital, National Center for Cardiovascular Diseases, Chinese Academy of Medical Sciences, Peking Union Medical College, Beijing, China; ^2^Animal Experimental Center, Beijing Key Laboratory of Pre-Clinical Research and Evaluation for Cardiovascular Implant Materials, State Key Laboratory of Cardiovascular Disease, Beijing, China

**Keywords:** myocardial perfusion, computed tomography, cardiac imaging techniques, radiation dosage, image quality enhancement

## Abstract

**Background:**

It is unclear whether tube voltage influences the measurement of perfusion parameters. The present study sought to evaluate the influence of tube voltage change on myocardial blood flow (MBF) measurements in dynamic computed tomography myocardial perfusion imaging (CTP).

**Methods and Results:**

Seven swine [mean weight 55.8 kg ± 1.6 (standard deviation)] underwent rest and stress dynamic CTP with tube voltages of 100 and 70 kV. The image noise, signal-to-noise ratio (SNR), contrast-to-noise ratio (CNR), radiation dose and MBF value were compared. The 70 kV images had higher CT attenuation and higher image noise (27.9 ± 2.4 vs. 21.5 ± 1.9, *P* < 0.001) than the 100 kV images, resulting in a higher SNR (20.5 ± 1.6 vs. 15.6 ± 1.8, *P* < 0.001) and CNR (17.6 ± 1.5 vs. 12.4 ± 1.7, *P* < 0.001). Compared to the use of conventional 100 kV, 70 kV yielded an approximately 64.6% radiation dose reduction while generating comparable MBF values, both at rest (88.3 ± 14.9 ml/100 g/min vs. 85.6 ± 17.4 ml/100 g/min, *P* = 0.21) and stress (101.4 ± 21.5 ml/100 g/min vs. 99.6 ± 21.4 ml/100 g/min, *P* = 0.58) states.

**Conclusion:**

Dynamic CTP using 70 kV instead of 100 kV does not substantially influence the MBF value but significantly reduces the radiation dose. Additional research is required to investigate the clinical significance of this change.

## Introduction

Coronary computed tomographic angiography (CCTA) has been validated as a noninvasive imaging modality to assess the degree of coronary artery stenosis (1) but is insufficient to provide functional assessment. However, functional assessment is important for the diagnosis and treatment of ischemic heart disease (2). Current diagnosis and treatment strategies recommend assessing myocardial function before revascularization (3).

Preliminary animal and clinical studies have shown that dynamic computed tomography myocardial perfusion imaging (CTP) may serve as a useful adjunct to CCTA to improve the specificity of detecting myocardial ischemia (4–7). In a recent meta-analysis, the diagnostic specificity of noninvasive evaluations to detect functionally significant coronary lesions for stress CTP added to CCTA was as high as 91%, using fractional flow reserve as the reference standard (8). Furthermore, similar to positron emission tomography imaging, dynamic CTP imaging offers the ability to obtain quantitative perfusion parameter data. However, the incremental radiation dose can occur due to its acquisition of repeated dynamic data. Specifically, the mean radiation dose ranged from 5.3 to 10.5 mSv per dynamic perfusion (9). The tube voltage determines the peak energy of the photon of a given X-ray energy spectrum, which impacts the dose more than other factors, such as tube current (10, 11). To limit radiation exposure, many clinical studies are already using lower tube voltage in their CTP protocols (7, 12).

Thus, we conducted an *in vivo* animal study by scanning the animal twice with both conventional (100 kV) and lower (70 kV) tube voltages while keeping the same tube current to evaluate the influence of tube voltage on the image quality, radiation dose, and MBF value.

## Materials and Methods

### General Methods

This study was approved by the local Institutional Animal Care and Use Committee, with Ethical Approval Number 0102-1-7-ZX(X)-21. Seven male domestic swine, 5–6 months old, with a mean body weight of 55.8 kg ± 1.6 were included in this study.

Animals were sedated with an intramuscular injection of ketamine hydrochloride (8–9 mg/kg, Zhongmu Beikang Pharmaceutical, Jiangsu, China) and xylazine hydrochloride (2 mg/kg, Changsha Best Biological Technology Institute, Hunan, China). Sedation maintenance was performed by continuous intravenous infusion of propofol (Fresenius Kabi Austria GmbH, Graz, Austria) at 2–3 mg/kg/h. Intravenous access was obtained in the right and left marginal auricular veins for the administration of propofol and adenosine triphosphate (ATP), respectively.

### Computed Tomography Protocol and Image Reconstruction

All swine were scanned on a wide-coverage, 256-row (16 cm in *z*-axis) CT scanner (Revolution CT, GE Healthcare, Milwaukee, WI, United States) with a tube rotation time of 0.28 s and a half-scan reconstruction algorithm was employed. The *z*-axis coverage was selected as 140 mm which is sufficient to cover the animal myocardium. The examination table did not move during the scanning. Prospective electrocardiogram (ECG)-gated scanning was performed using a dynamic acquisition protocol at the end-systolic phase (45% R-R interval) and a visual bolus tracking method was used. For contrast agent and saline, a standard 20-gauge intravenous catheter was inserted into the anterior cubital vein.

Each animal was scanned using two protocols both at rest and stress states. For the conventional-dose protocol (Protocol 1) tube voltage was set at 100 kV, and tube current was set at 200 mA. For the low-dose protocol (Protocol 2), the tube voltage was set at 70 kV, and the tube current was fixed at 200 mA. Scanning was completed within 1 h of the completion of sedation for each swine. Both rest and stress scans were performed successively in one protocol with a 30-min interval. Protocol 1 and Protocol 2 shared identical rest and stress scan protocols. There was a 5-day interval between Protocol 1 and Protocol 2 with a random scanning order.

Iopamidol (Beijing Beilu Pharmaceutical, Beijing, China) with an iodine concentration of 350 mgI/100 ml was injected at 2.5 ml/s (for 8 s). After the contrast agent was injected, 20 ml of saline flush was injected at a rate of 4.5 ml/s. For the scans at the rest state, scanning would initiate when contrast media arrived at the superior vena cava followed by a delay of 1.7 s between the scan trigger time and the actual scan start time. Three consecutive phases of datasets were acquired covering the base of the myocardium to the apex with a total acquisition time of approximately 25 s (Table 1). For the scans at the stress state, the contrast media and scanning were initiated 3 min after administration of intravenous ATP (Sinopharm, Henan, China) injection at 140 μg/kg/min. The scan timing was the same as the rest state. The heart rates were continuously monitored.

**TABLE 1:** Dynamic myocardial perfusion acquisition.

	Phase 1	Phase 2	Phase 3
kV Mode	100/70	100/70	100/70
mA Mode	200 mA	200 mA	200 mA
Prep/Phase delay	0 s	1.10 s	1.80 s
Detector coverage	140 mm	140 mm	140 mm
Number of passes	13	3	2
Minimum time between passes	0.80 s	1.40 s	5.00 s
rotation time	0.28 s	0.28 s	0.28 s
Total exposure time*	3.96 s	0.92 s	0.61 s
Primary Recon thickness	1.25 mm	1.25 mm	1.25 mm
Reconstruction kernel	Standard	Standard	Standard
ASIR-V	100%	100%	100%

*Heart rate at 77 bpm; ASIR-V, adaptive statistical iteration reconstruction-V.

All datasets of rest and stress images were transferred to a dedicated postprocessing workstation (Advantage Workstation Version 4.7, GE Healthcare). A proprietary three-dimensional non-rigid image registration algorithm was used to minimize the misalignment of dynamic contrast enhancement images arising from residual cardiac and respiratory motion among scans during perfusion imaging. A dedicated myocardial perfusion software package (CT perfusion 4D, GE Healthcare) was used for myocardial perfusion analysis.

### Objective Assessment of Image Quality, Enhancement of Myocardium, and Radiation Dose

One cardiovascular radiologist who had more than 5 years of clinical experience in cardiac imaging evaluated the objective image quality of the two protocols (both at rest and stress). The time point of peak enhancement in the ascending aorta was determined. The axial images with a 1.25 mm slice thickness was selected for image quality evaluation. For myocardium attenuation, a region of interest (ROI) was chosen to be as large as the myocardial septal thickness. The attenuation of the left ventricle cavity was measured using a 2.0 cm^2^ ROI in a homogeneous region without obvious papillary muscle. Image noise was measured using the standard deviation (SD) of the pixel values on ventricular septal (13). The signal-to-noise ratio (SNR) and contrast-to-noise ratio (CNR) are described in Eqs. 1 and 2, respectively.


(1) $$\text{SNR}=\frac{\mathrm{Attenuation}\,\mathrm{of}\,\mathrm{Left}\,\mathrm{ventricle}\,\mathrm{cavity}\,\left(\mathrm{HU}\right)}{S\,D}$$

(2) $$\mathrm{CNR}=\frac{\mathrm{Attenuation}\,\mathrm{of}\,\mathrm{LV}\,\mathrm{cavity}\,\left(\mathrm{HU}\right)-\mathrm{Attenuation}\,\mathrm{of}\,\mathrm{myocardium}\,\left(\mathrm{HU}\right)}{\mathrm{SD}}$$

Furthermore, the baseline and peak enhancement of the myocardium were measured to evaluate the enhancement of myocardium. The relative enhancement of the myocardium is the difference between the baseline and peak enhancement. Beam-hardening artifacts were also evaluated, which were considered hypoattenuated areas.

To assess the radiation dose, the dose length product (DLP, mGy-cm) was recorded from the study dose summary. The radiation doses and image quality were compared by protocols.

### Myocardial Blood Flow Calculation

**FIGURE 1:**
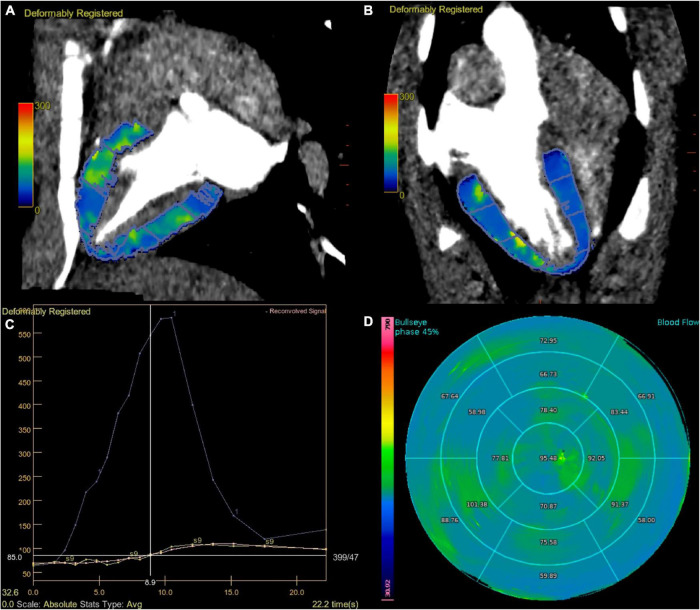
Dynamic perfusion acquisition at 70 kV. Fused MBF map and CT images, divided according to AHA segments **(A,B)**. The graph view displays the AIF (displayed in blue), TAC of the myocardium (displayed in yellow), and reconvolution curve (displayed in pink). A smooth fitting curve (pink line) was obtained through the TAC of each segmental myocardium (yellow line) **(C)**. Bull’s-eye map with quantitative MBF values, based on 70 kV of stress state **(D)**. AHA, American Heart Association; AIF, artery input function; TAC, time attenuation curve.

Another one cardiovascular radiologist who had more than 5 years of clinical experience calculated MBF. A region of interest (ROI) was placed in the ascending aorta to obtain the arterial input function (AIF). The time attenuation curves (TACs) of the myocardium were automatically identified and then manually edited if identification errors existed, and other procedures were automatically generated. Using a deconvolution model as described before (14), the MBF maps based on the 17-segment AHA myocardial model and mean MBF value of each segment were calculated automatically (Figure 1). Each of the MBF calculations was performed twice by the same reader to assess the intraobserver reliability. Data generated from the segments affected by beam-hardening artifacts were not included in the analysis.

### Statistical Analysis

Statistical analysis was performed using MedCalc 19.0.4 (MedCalc, Mariakerke, Belgium). The intraobserver reliability of MBF was assessed by calculating the intraclass correlation coefficient (ICC). All continuous data are expressed as the means ± standard deviations. For the test of normality, the Shapiro–Wilk test was used, and two groups were compared using paired Student’s *t*-test. *P* < 0.05 was considered statistically significant. In addition to quantifying the strength of the differences, Cohen’s *d* was calculated as a measure of effect size (*d* = 0.2 represents a “small” effect size, 0.5 represents a “medium” effect size, and 0.8 a “large” effect size).

## Results

### General Results

All seven swine completed the study without complications. Each swine was scanned for Protocol 1 and Protocol 2 both at rest and in the stress state, and a total of 28 data sets were obtained. Among the 476 myocardial segments, 19 (4.0%) were considered unevaluable because of beam-hardening artifacts. The mean increase in heart rate after stress was 10.2 ± 2.3 (range 6–15). There was no significant difference in the rest heart rate (75.9 ± 9.9 vs. 72.0 ± 11.3, *P* = 0.30) or stress heart rate (85.1 ± 9.0 vs. 81.6 ± 10.1, *P* = 0.86) between Protocol 1 (100 kV) and Protocol 2 (70 kV) (Table 2).

**TABLE 2:** Summary of the heart rate and perfusion results.

	Protocol 1 (100 kV)	Protocol 2 (70 kV)	*P*-value	Effect size
Heart rate-rest	75.9 ± 9.9	73.7 ± 13.1	0.57	0.14
Heart rate-stress	85.1 ± 9.0	84.1 ± 10.8	0.78	0.11
MBF rest (ml/100 g/min)	88.3 ± 14.9	85.6 ± 17.4	0.21	0.13
MBF stress (ml/100 g/min)	101.4 ± 21.5	99.6 ± 21.4	0.58	0.06

*MBF, myocardial blood flow.*

### The Results of Objective Evaluation

**FIGURE 2:**
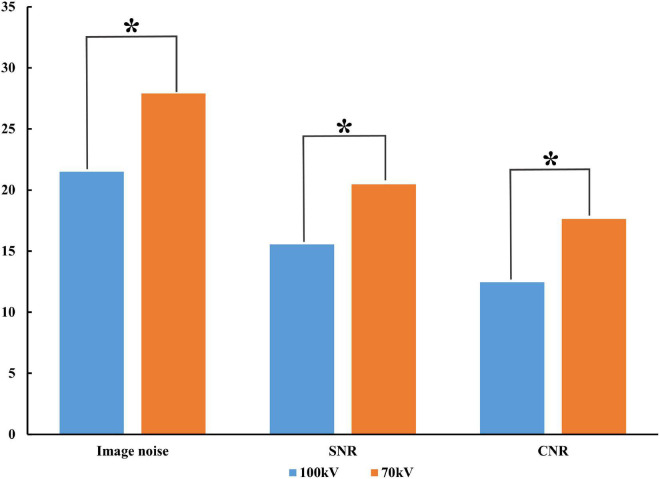
Comparison of image quality between protocols. Compared to 100 kV, 70 kV image noise increased along with SNR and CNR. SNR, signal-to-noise ratio; CNR, contrast-to-noise ratio; *P < 0.001.

**FIGURE 3:**
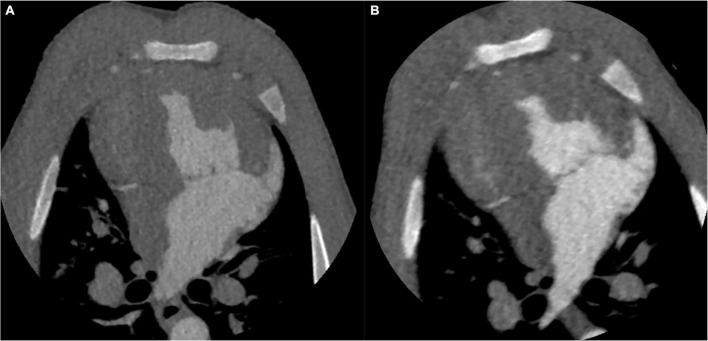
CT images of the identical swine at different tube voltages. CT images on the aortic peak enhancement of the dynamic series (window width = 1200, window level = 240). The CT image at 100 kV. The image noise, SNR and CNR were 20.8, 16.2, and 12.9, respectively **(A)**. The CT image at 70 kV. The image noise, SNR and CNR were 27.7, 20.6, and 17.9, respectively **(B)**. SNR, signal-to-noise ratio; CNR, contrast-to-noise ratio.

The image with 70 kV showed higher image noise and higher attenuation. The mean noise measured at 70 kV was 29.8% higher than that measured at 100 kV (27.9 ± 2.4 vs. 21.5 ± 1.9, *P* < 0.001). There was a higher SNR (20.5 ± 1.6 vs. 15.6 ± 1.8, *P* < 0.001) and CNR (17.6 ± 1.5 ± 4.3 vs. 12.4 ± 1.7, *P* < 0.001) at 70 kV than at 100 kV (Figure 2). There was a nearly 64.6% radiation reduction in the 70 kV protocol compared with the 100 kV protocol (mean DLP, 123.4 ± 0.2 vs. 348.3 ± 0.7 mGy-cm; *P* < 0.001). Likewise, the baseline, peak enhancement and relative enhancement of the myocardium were higher at 70 kV than at 100 kV (Table 3). Representative images of image quality are presented in Figure 3.

**TABLE 3:** The results of objective evaluation.

	Protocol 1 (100 kV)	Protocol 2 (70 kV)	*P*-value	Effect size
Attenuation of the LV cavity (HU) at peak enhancement of ascending aorta	333.2 ± 39.6	570.2 ± 61.6	< 0.001	2.81
Baseline attenuation of myocardium (HU)	56.4 ± 5.0	66.8 ± 2.8	< 0.001	2.10
Attenuation of the myocardium (HU) at peak enhancement of ascending aorta	66.8 ± 7.5	77.9 ± 10.6	0.008	0.85
Peak enhancement of myocardium (HU)	83.9 ± 3.6	100.2 ± 4.4	< 0.001	2.81
Relative enhancement of the myocardium	27.6 ± 4.4	31.4 ± 3.4	0.04	0.60
Image noise (HU)	21.5 ± 1.9	27.9 ± 2.4	< 0.001	2.00
SNR	15.6 ± 1.8	20.5 ± 1.6	< 0.001	1.68
CNR	12.4 ± 1.7	17.6 ± 1.5	< 0.001	2.11
DLP (mGy-cm)	348.3 ± 0.7	123.4 ± 0.2	< 0.001	282.93

*LV, left ventricle; HU, Hounsfield unit; SNR, signal-to-noise ratio; CNR, contrast-to-noise ratio; DLP, dose-length product.*

### Myocardial Blood Flow Comparison

**FIGURE 4:**
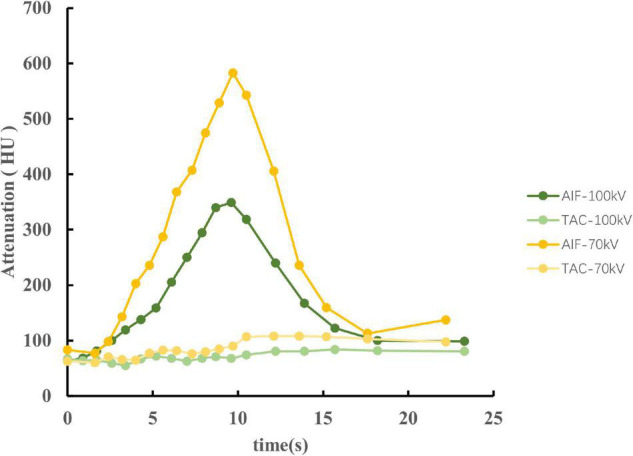
The AIF of the ascending aorta and TACs of the myocardium between 100 and 70 kV in an identical swine of stress state. The CT attenuation was higher at 70 kV, causing a large slope of the AIF. Therefore, the AIF of 70 kV has a sharp peak compared to 100 kV. However, both AIF and TAC showed similar trends between 70 and 100 kV. AIF, arterial input functions; TACs, time attenuation curves.

**FIGURE 5:**
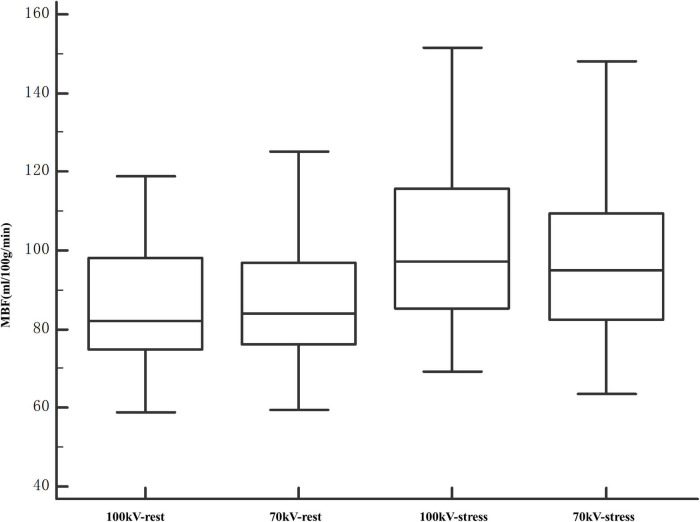
Comparison of MBF values at segment levels of 100 and 70 kV. The entire range of measured MBF values increased after stress. The MBF values were comparable between 100 and 70 kV in both rest and stress states. MBF, myocardial blood flow.

Arterial input function of the ascending aorta and TACs of the myocardium between the two protocols are illustrated in Figure 4. The reproducibility of the MBF calculation was excellent, with an intraobserver intraclass correlation coefficient of 0.93 (95% confidence interval, 0.87–0.95). There was no significant difference between 100 and 70 kV in the MBF at the segmental level, either at the rest (88.3 ± 14.9 ml/100 g/min vs. 85.6 ± 17.4 ml/100 g/min, *P* = 0.21) or at the stress (101.4 ± 21.5 ml/100 g/min vs. 99.6 ± 21.4 ml/100 g/min, *P* = 0.58) state (Table 2 and Figure 5).

## Discussion

In this study, we assessed the influence of tube voltage on the image quality, radiation dose and quantification of MBF based on swine scans. Specifically, we illustrated that compared with 100 kV, 70 kV obtained higher CT attenuation and image noise, resulting in a higher SNR and CNR. Notably, the low-dose scanning protocol had a nearly 64.6% dose reduction and a comparable MBF.

The animal model used in this study simulated the human condition most closely because the swine weighed an average of 55.8 kg, and their heart and vessel sizes were comparable to those of humans. The MBF values increased less than that of previous human study of normal myocardium after stress (6). This may be caused by the following reasons. First, the animals in this study were under sedation, which may reduce MBF. To minimize the sedation effect, all swine were scanned randomly within 1 h of the sedation completion. Second, the dosage of ATP is based on clinical experience (7, 15). For animals in a sedated state, the dosage of ATP used in the present study may not allow the animals to reach the maximum vasodilated state. However, the MBF comparison was carried out in the same state, which means that the above factors do not affect the conclusion.

Several clinical studies have shown that MBF is a useful parameter to differentiate normal and ischemic myocardium (6, 12). One of the limitations of dynamic CTP has been the acquisition of multiple consecutive phases, which may result in a higher absorbed radiation dose (16). Two clinical studies regarding dual-source dynamic myocardial CTP investigated tube voltage and current on the image quality and MBF using a clinically matched cohort (17, 18). According to their research, reducing the tube voltage combined with changing the tube current was associated with a significant reduction in the mean radiation dose while obtaining a comparable MBF value, which was consistent with our results. In contrast to their studies, instead of clinically matched cohorts, a homogeneous group of swine was scanned twice with only the tube voltage changed, making the measurements more compatible.

Iodine shows a characteristic CT attenuation spectrum with high attenuation at low photon energies (19, 20), which allows differentiation from other materials and mapping tissue iodine distribution as a surrogate for tissue perfusion. The increased intravascular contrast allows for higher image noise at a lower tube potential, thus reducing the radiation dose without deteriorating image quality. In the present study, we reduced the tube voltage to 70 kV with a fixed tube current for dose reduction and obtained higher CT attenuation and image noise, as expected, resulting in a higher SNR and CNR, which has been demonstrated previously in a clinical study (21). Skornitzke et al. reported that an increase in image noise leads to an increase in MBF measurements in digital perfusion phantoms (22). However, only the effect of image noise on TACs was considered in their study. In addition, only one AIF was evaluated at a time. In other words, the tube voltage was fixed, which inevitably caused deterioration in the SNR and CNR. In the present study, with an increase in image noise, the SNR and CNR also increased, which may be attributed to the comparable MBF. Meanwhile, the higher CT attenuation at low tube voltage results in a large slope of the AIF. Therefore, the AIF of 70 kV has a sharp peak compared to 100 kV (Figure 4). However, the results of this study suggest that it does not seem to substantially influence the MBF value.

Although image noise levels may vary among protocols on different CT scan devices, the relationship between noise and image quality (Eqs. 1 and 2) should continue to hold true. Our study suggests that the MBF value between the two protocols may be comparable. Although further validation is still needed, this result may have important implications in clinical research. Our study supports the possibility that dose reduction can sometimes be achieved with a reduction in tube voltage alone.

There are some limitations in our study. First, the number of animals included in this study was relatively small. However, MBF was calculated for multiple segments, resulting in the evaluation of nearly 238 segments per scan protocol. Second, a “visual bolus tracking” method was used to minimize the radiation dose while collecting baseline data sets. Based on our research, generally, 2–3 baseline data sets were obtained. The reduced baseline data acquisition might affect the MBF calculation. However, the baseline acquisition within the research was consistent. All data acquisitions were obtained using the same protocol except for the change in tube voltage. Third, there was just 30 min between the rest and stress, which has the potential for residual contrast in the system.

## Conclusion

Dynamic CTP using 70 kV instead of 100 kV does not substantially influence the MBF value while significantly reducing the radiation dose. For the clinical application of dynamic CTP, low-dose modes based on low tube voltage scanning may be considered in dose-reduction strategies. Future studies should investigate the clinical significance of this change.

## Data Availability Statement

The original contributions presented in the study are included in the article/supplementary material, further inquiries can be directed to the corresponding author/s.

## Ethics Statement

The animal study was reviewed and approved by the Chinese Academy of Medical Sciences, Fuwai Hospital, Institutional Animal Care and Use Committee (IACUC).

## Author Contributions

WG and BL contributed to the conception and design of the study. WG, HY, NZ, LJ, and WM collected the data. WG and YA performed the statistical analysis. WG drafted the manuscript. YG and BL revised the manuscript critically. All authors contributed to manuscript revision, read, and approved the submitted version.

## Conflict of Interest

The authors declare that the research was conducted in the absence of any commercial or financial relationships that could be construed as a potential conflict of interest.

## Publisher’s Note

All claims expressed in this article are solely those of the authors and do not necessarily represent those of their affiliated organizations, or those of the publisher, the editors and the reviewers. Any product that may be evaluated in this article, or claim that may be made by its manufacturer, is not guaranteed or endorsed by the publisher.
